# Giant retroperitoneal leiomyosarcoma: a case report

**DOI:** 10.1093/jscr/rjad172

**Published:** 2023-04-12

**Authors:** Amine Hermi, Hamza Boussaffa, Ahmed Saadi, Linda BelHadjKacem, Marouene Chakroun, Riadh Ben Slama

**Affiliations:** Department of Urology, Charles Nicolle Hospital, University Tunis Manar, Faculty of Medicine Tunis, Tunis 1001, Tunisia; Department of Urology, Charles Nicolle Hospital, University Tunis Manar, Faculty of Medicine Tunis, Tunis 1001, Tunisia; Department of Urology, Charles Nicolle Hospital, University Tunis Manar, Faculty of Medicine Tunis, Tunis 1001, Tunisia; Department of Pathology, Charles Nicolle Hospital, University Tunis Manar, Faculty of Medicine Tunis, Tunis 1001, Tunisia; Department of Urology, Charles Nicolle Hospital, University Tunis Manar, Faculty of Medicine Tunis, Tunis 1001, Tunisia; Department of Urology, Charles Nicolle Hospital, University Tunis Manar, Faculty of Medicine Tunis, Tunis 1001, Tunisia

**Keywords:** sarcoma, retroperitoneal, leiomyosarcoma

## Abstract

Retroperitoneal leiomyosarcomas are rare tumors, mostly malignant. They are silent slow growing, and at the time of diagnosis, they are often of a considerable size. Management necessitates en bloc resection of the mass with adjacent organs, which is often challenging due to large size of the tumor. Herein, we present a case of 59-year-old male patient presenting for surgical management of 190 × 150 × 140 mm retroperitoneal leiomyosarcoma.

## INTRODUCTION

Retroperitoneal leiomyosarcomas (RPL) are rare tumors of the mesenchymal cells originating from nerve, muscular or vascular tissues [1]. It accounts less than 1 in 100 000 of all malignancies [2]. It is the second most frequent type of retroperitoneal sarcomas (28%), after liposarcoma, which has the best prognosis [1].

It remains clinically silent given the anatomical adaptability of the retroperitoneum. It is often diagnosed incidentally, being asymptomatic, at a considerable size with close proximity to adjacent organs [2]. The mainstay of treatment for RPL is complete surgical resection whenever possible. As these tumors are often massive in size, surgery can be challenging.

## CASE REPORT

**Figure 1:**
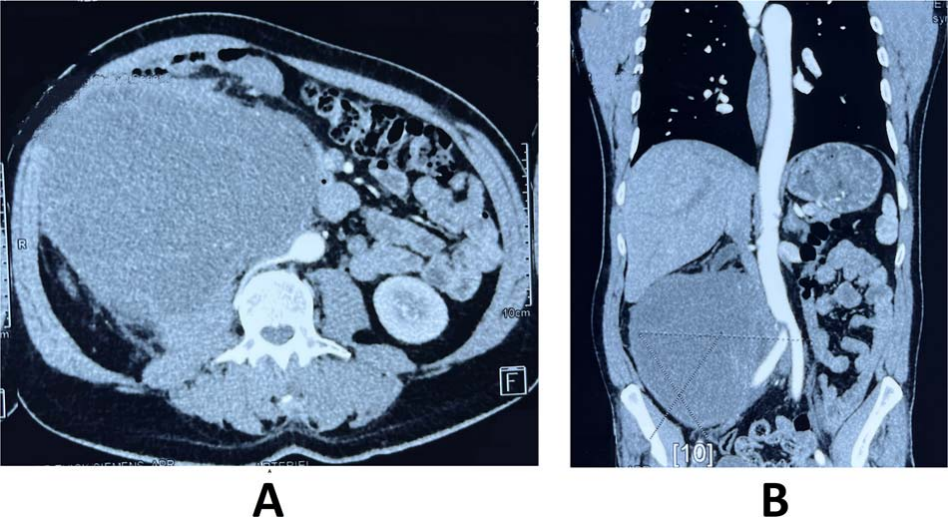
Axial (**A**) and frontal (**B**) view of Computed tomography scan showing a large right retroperitoneal solid mass, with lobulated thickened wall measuring 190 × 140 × 120 mm, heterogeneously enhancing, displacing anteriorly duodenal loops, laterally the right colon, medially the inferior vena cava and abdominal aorta.

A 59-year-old male patient, known to have hypertension controlled by medication with no previous abdominal surgeries or any remarkable family history, presented with the complaint of a palpable indolent abdominal mass and vague abdominal fullness evolving for 4 months. The patient denied gross hematuria and lumbar pain. Clinical examination showed, on inspection, a visible large abdominal mass occupying the right hemiabdomen. The mass was hard and fixed to deeper layers. Neither jaundice nor dilated skin veins was found. Lab tests were normal. A thoraco-abdominal-pelvic computed tomography with contrast medium and delayed phases was performed. It showed a large right retroperitoneal solid mass measuring 190 × 140 × 120 mm with a thickened lobulated wall. It was heterogeneously enhancing, managing hypoattenuating areas, compatible with necrosis. It was responsible for anterior displacement of intestinal loops and pushed laterally the right colon. It crossed the midline displacing medially the inferior vena cava, abutting the abdominal aorta. Right mild hydronephrosis was found by direct invasion of the ureter. No clear dependence of any of the adjacent organ was identified. No metastatic lesions were found (Fig. 1).

Tumors markers (CA-125, Carcino-Embryonic-Antigen and alpha-foetoprotein) were normal.

A scan-guided biopsy of the mass was realized. It was non-contributory showing necrotic tissue without specific cells.

**Figure 2:**
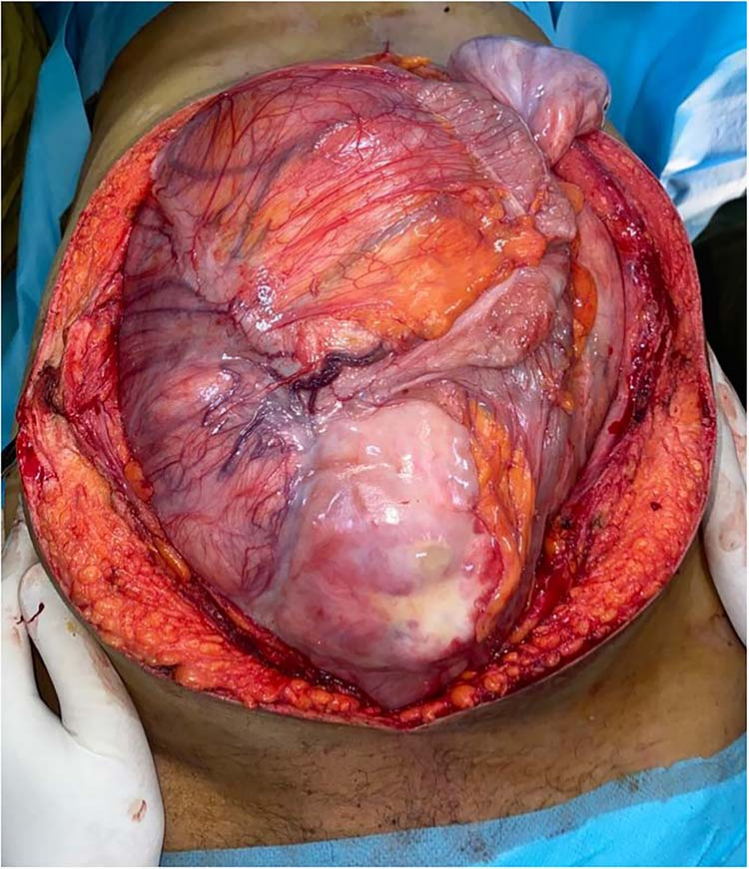
Preoperative view of the large lobulated mass occupying largely the abdominal cavity.

Surgery was performed by a midline xipho-pubic incision. The tumor was occupying the entire abdominal cavity, involving right lumbar ureter, and terminal ileum. Meticulous dissection was done and the mass was freed from adherences. After confirming the resectability of the tumor, we realized complete resection of the mass, a 20-centimeter invaded ileum loop with right kidney and ureter. Immediate ilio-ileal anastomosis was realized. No vascular repair was needed. Operative specimens weighted 16 kilograms (Fig. 2). Patient had a smooth postoperative course and was discharged home 4 days after surgery.

**Figure 3:**
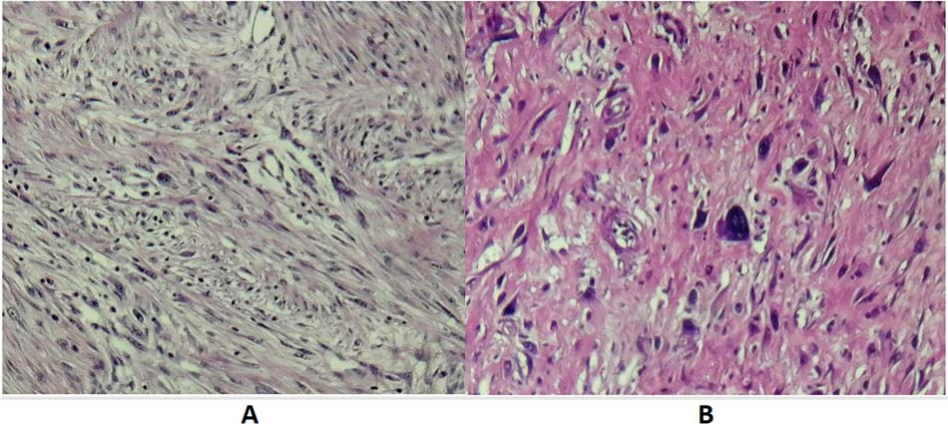
(**A**) Microscopic examination showing spindle cells with elongated hyperchromatic nuclei and arranged in interlacing bundles of (HE X 200). (**B**) Tumor cells showing nuclear atypia and eosinophilic cytoplasm (HE X 400).

Histopathological examination of operative specimens showed focal areas of necrosis accounting for less than 50% of the tumor mass. Tumor cells were spindle shaped, pleomorphic and had moderate to severe atypia. Mitotic figures were abundant, counting 1–2 mitosis per 10 CFG. Surgical margins were free of disease. Renal parenchyma, renal pelvis and ureter were free of neoplasia. Pathology turned out to be RPL Grade II according to the French staging system for sarcoma tumors FNLCC (Fig. 3).

The patient’s case was discussed at the tumor board. The decision was, due to negative surgical margin, that the patient should be followed by clinical and radiological exams, without adjuvant chemotherapy or radiotherapy. On 1-year follow-up, the patient is disease free with no complaint.

## DISCUSSION

RPL are slow growing tumors, diagnosed incidentally in asymptomatic patients or with nonspecific symptoms [3], usually secondary to the mass effect of the large tumor, such as early satiety or abdominal distension. Rare cases of paraneoplastic hypoglycemia were reported (insulin-like growth factor 2) [4].

Complete resection of RPL with intact capsule is the cornerstone of the management of this entity. En bloc resection of tumor with adjacent organ resection, most commonly the kidney and colon, is frequently needed to achieve complete resection [4]. It is the most important feature to improve the overall survival rate. Studies have shown that the median survival of patients with complete resection was 103 months versus 18 months with incomplete resection, similar to non-operative treatment outcome [2].

Various types of chemotherapeutic agents, such as anthracycline-based chemotherapy: Doxorubicin, have been utilized over the years in an attempt to increase survival. It has not demonstrated to increase survival neither provide a better quality of life [2]. Preoperative chemotherapy, although not common, is an acceptable alternative, but it is considered a category 2B recommendation of the National Comprehensive Cancer Netrwork (NCCN) guidelines 2022 [5].

The potential benefit of neoadjuvant radiation includes decreasing tumor size, improving resectability, and improving local control. No studies have definitely demonstrated a benefit to preoperative radiation for RPL [5]. Some studies reported that the 5-year disease-free survival was 37% with an overall survival of 56% in patients treated with a dose of 50.4 Gy [5].

Similar to chemotherapy, no randomized controlled trials of adjuvant radiotherapy have been published, but many clinicians advocate the use of radiotherapy in patients found to have positive margins. Patients who were given doses of radiation between 53 and 56.4 Gray after undergoing surgical resection with varying results. Preoperative radiation is a category 2B recommendation of the NCCN, for tumors at high risk for local recurrence [5].

In patients undergoing an R0 resection, postoperative radiotherapy should be given to patients with high-grade tumors, extremely large tumors, and those with close margins. After an R1 resection, the NCCN recommends postoperative radiotherapy if neoadjuvant therapy was not given and a boost of 10–16 Gy if preoperative radiotherapy was given [5]. In case of locoregional recurrence (20–75% of patients, even if R0), surgery still remains the mainstay of treatment in the absence of distant metastatic disease [4].

It is often more complicated. Multiple studies have also shown that the metastatic progression of the disease is associated with significantly lower rates for complete resection (70%). In case of unresectability, radiation therapy (if unifocal recurrence) or systemic therapy can be proposed [4].

## CONCLUSION

RPL is a rare tumor, mostly malignant. The best therapeutic option remains the complete surgical resection with negative microscopic margins, often challenging. A multidisciplinary approach is highly advocated to improve overall survival rate.
